# Single-subunit oligosaccharyltransferases of *Trypanosoma brucei* display different and predictable peptide acceptor specificities

**DOI:** 10.1074/jbc.M117.810945

**Published:** 2017-09-19

**Authors:** Anders Jinnelov, Liaqat Ali, Michele Tinti, Maria Lucia S. Güther, Michael A. J. Ferguson

**Affiliations:** From the Wellcome Centre for Anti-Infectives Research, School of Life Sciences, University of Dundee, Dundee DD1 5EH, Scotland, United Kingdom

**Keywords:** glycoprotein biosynthesis, N-linked glycosylation, oligosaccharyltransferase, parasitology, Trypanosoma brucei, STT3, glycoproteomics, machine learning

## Abstract

*Trypanosoma brucei* causes African trypanosomiasis and contains three full-length oligosaccharyltransferase (OST) genes; two of which, *Tb*STT3A and *Tb*STT3B, are expressed in the bloodstream form of the parasite. These OSTs have different peptide acceptor and lipid-linked oligosaccharide donor specificities, and trypanosomes do not follow many of the canonical rules developed for other eukaryotic *N*-glycosylation pathways, raising questions as to the basic architecture and detailed function of trypanosome OSTs. Here, we show by blue-native gel electrophoresis and stable isotope labeling in cell culture proteomics that the *Tb*STT3A and *Tb*STT3B proteins associate with each other in large complexes that contain no other detectable protein subunits. We probed the peptide acceptor specificities of the OSTs *in vivo* using a transgenic glycoprotein reporter system and performed glycoproteomics on endogenous parasite glycoproteins using sequential endoglycosidase H and peptide:*N*-glycosidase-F digestions. This allowed us to assess the relative occupancies of numerous *N*-glycosylation sites by endoglycosidase H-resistant *N*-glycans originating from Man_5_GlcNAc_2_-PP-dolichol transferred by *Tb*STT3A, and endoglycosidase H-sensitive *N*-glycans originating from Man_9_GlcNAc_2_-PP-dolichol transferred by *Tb*STT3B. Using machine learning, we assessed the features that best define *Tb*STT3A and *Tb*STT3B substrates *in vivo* and built an algorithm to predict the types of *N*-glycan most likely to predominate at all the putative *N*-glycosylation sites in the parasite proteome. Finally, molecular modeling was used to suggest why *Tb*STT3A has a distinct preference for sequons containing and/or flanked by acidic amino acid residues. Together, these studies provide insights into how a highly divergent eukaryote has re-wired protein *N*-glycosylation to provide protein sequence-specific *N*-glycan modifications. Data are available via ProteomeXchange with identifiers PXD007236, PXD007267, and PXD007268.

## Introduction

The tsetse-fly–transmitted protozoan parasite *Trypanosoma brucei* and its close relatives are responsible for human and animal African trypanosomiasis. The animal-infecting bloodstream forms of these organisms depend on surface coats made of glycosylphosphatidylinositol (GPI)^3^-anchored and *N-*glycosylated variant surface glycoprotein (VSG) to evade the innate host immune system (1) and the acquired immune system through antigenic variation (2). Furthermore, they express many less abundant glycoproteins such as their novel transferrin receptors (3–5), a novel lysosomal/endosomal protein called p67 (6), the so-called invariant surface glycoproteins (ISGs) (7), and invariant endoplasmic reticulum glycoproteins (8), the Golgi/lysosomal glycoprotein tGLP-1 (9), the membrane-bound histidine acid phosphatase TbMBAP1 (10), the flagellar adhesion zone glycoproteins Fla1 and Fla2 (11), and others. Whereas some of these are bloodstream-form specific glycoproteins (VSGs, ISGs, *Tb*MBAP1, and transferrin receptors), others are common to the tsetse midgut-dwelling procyclic form of the parasite. Furthermore, procyclic form parasites also express unique glycoproteins, notably the abundant GPI-anchored procyclins, some of which are *N*-glycosylated (12, 13), and the partially characterized high-molecular-weight glycoconjugate (14, 15). Many of the *N-*glycan structures expressed by *T. brucei* have been solved, and these include conventional oligomannose and biantennary complex structures as well as paucimannose and extremely unusual “giant” poly-*N-*acetyl-lactosamine (poly-LacNAc) containing complex structures in the bloodstream form of the parasite (16–19). In contrast, only oligomannose *N*-glycans have been structurally described in wild-type procyclic trypanosomes (12, 20). The unusual repertoire of the *T. brucei* bloodstream-form *N-*glycans and the original observation by Bangs *et al.* (21) that Endo H-resistant *N-*glycans appear immediately following protein synthesis, and not following transport to the Golgi apparatus, has stimulated our group to study the fundamentals of protein *N-*glycosylation in this divergent eukaryotic pathogen.

Protein *N-*glycosylation is believed to be a ubiquitous post-translational modification among the eukaryotes, with the canonical model based primarily on extensive studies in mammalian cells and the yeast *Saccharomyces cerevisiae* (22, 23). In this canonical model, there are a number of tenets that include: (i) The mature lipid-linked oligosaccharide (LLO) donor, and the preferred substrate for oligosaccharyltransferases (OSTs) is Glc_3_Man_9_GlcNA_2_-PP-dolichol. (ii) The OSTs are hetero-oligomers of 8 or 9 distinct subunits. (iii) The OSTs may fall into two classes (A and B) according to their subunit composition with different peptide acceptor specificities. (iv) The ER enzyme UDP-glucose–glycoprotein glucosyltransferase (UGGT) operates on tri-antennary Man_9–7_GlcNA_2_ structures with a complete a-branch, but not on bi-antennary structures. (v) The action of Golgi mannosidase II is a pre-requisite for the conversion of oligomannose to complex *N-*glycans. (vi) The enzymes GnTI and GnTII have strict acceptor substrate specificities, operating on Man_5_GlcNA_2_ and GlcNAcMan_3_GlcNA_2_, respectively. However, none of these tenets apply to *N-*glycosylation in the protozoan parasite *T. brucei* where the following points have been noted. (i) The largest LLO is Man_9_GlcNA_2_ (24, 25) and different OSTs preferentially transfer either this structure or bi-antennary Man_5_GlcNA_2_ (25–28). (ii) There is no evidence for OST subunits other than the catalytic SST3 subunits in *T. brucei* or the related parasites *Trypanosoma cruzi* and the *Leishmania* (26, 28–32). (iii) OST subclasses and their peptide acceptor specificities (which are more disparate in *T. brucei* than for other eukaryotes) are defined only by their STT3 components (28). (iv) The parasite UGGT works efficiently on all structures (bi- and tri-antennary) with an intact a-branch (28). (v) Golgi-mannosidase II is absent, preventing the conversion form the oligomannose series to the complex series of *N-*glycans (25). (vi) The parasite GnTI and GnTII βGlcNAc-transferases have different specificities to canonical GnTIs and GnTIIs and belong to a different glucosyltransferase family (33, 34).

Here, we present the following. (i) We directly address the oligomeric states of *T. brucei* STT3 subunits. (ii) We look for evidence for any non-canonical OST subunits in addition to the *Tb*STT3s. (iii) We probe the peptide acceptor specificities of *Tb*STT3A (Tb927.5.890) and *Tb*STT3B (Tb927.5.900) using a reporter glycoprotein expression system and by glycoproteomics. (iv) We use machine learning to predict which putative *N*-glycosylation sites in bloodstream-form *T. brucei* will be modified by *Tb*STT3A or *Tb*STT3B.

## Results

### Blue native gel electrophoresis of in situ tagged TbSTT3A suggests it is present in high molecular weight complexes

**Figure 1.**
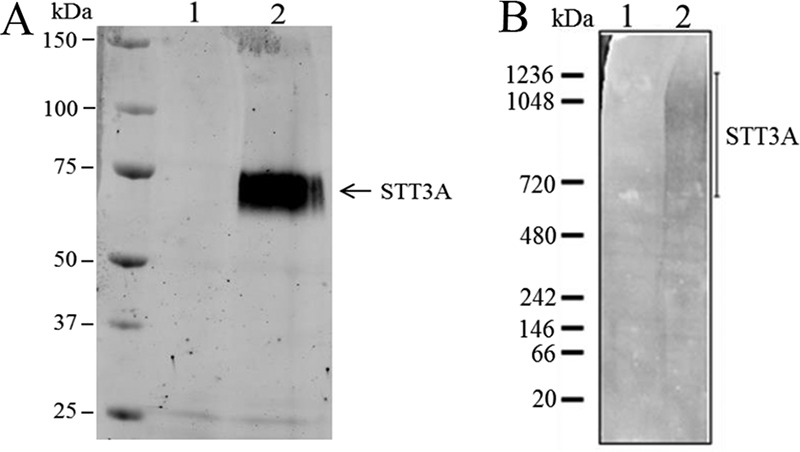
**Denaturing and blue native gel electrophoresis of *Tb*STT3A-HA3.** *A* and *B*, SDS-PAGE and anti-HA Western blotting (*A*) or blue native gel electrophoresis and anti-HA Western blotting (*B*) of anti-HA/protein G magnetic bead pull-outs from digitonin lysates of wild-type (*lane 1*) and *in situ Tb*STT3A-HA3-tagged (*lane 2*) bloodstream-form trypanosomes.

To enable immunoprecipitation of *Tb*STT3A, the 3′-end of the endogenous gene in a heterozygote cell line (*Tb*STT3A/B/C^+/−^) was tagged *in situ* with a sequence encoding a C-terminal HA_3_ epitope. Transfected cells were cloned and analyzed by Southern blotting to confirm correct insertion of the tag (supplemental Fig. S1*A*). To check that the tag did not impair the function of *Tb*STT3A, the glycosylation of VSG221 was analyzed in these cells. VSG221 receives different types of glycan at the two *N-*glycosylation sequons in the protein: Endo H-resistant Man_5_GlcNAc_2_ at Asn-263 and Endo H-sensitive Man_9_GlcNAc_2_ at Asn-428 (17, 28). Following PNGaseF and Endo H treatment, the typical digestion pattern for wild-type VSG221 was seen also for the transgenic cells (supplemental Fig. S1*B*), showing that C-terminally tagged *Tb*STT3A-HA_3_ is functional. Subsequently, cells were lysed under mild conditions (0.5% digitonin on ice for 30 min), and the clarified cell lysates were incubated with anti-HA mouse antibody, followed by magnetic beads coupled to protein G. The pull-out eluates were analyzed by SDS-PAGE and Western blotting using an anti-HA antibody. As expected, epitope-tagged *Tb*STT3A-HA_3_ was detected running just below the position of the 75-kDa molecular mass marker in the *Tb*STT3A-HA_3_ pull-out, whereas no band was seen in the wild-type cell pull-out (Fig. 1*A*). However, when the same eluates were analyzed by blue native gel electrophoresis and anti-HA Western blotting, a smear (specific for the *Tb*STT3A-HA_3_ cell line) was detected between 700 and 1200 kDa, suggesting that *Tb*STT3A is present in large complexes (Fig. 1*B*).

### SILAC proteomics shows TbSTT3A and TbSTT3B form hetero-oligomeric complexes without other subunits

**Figure 2.**
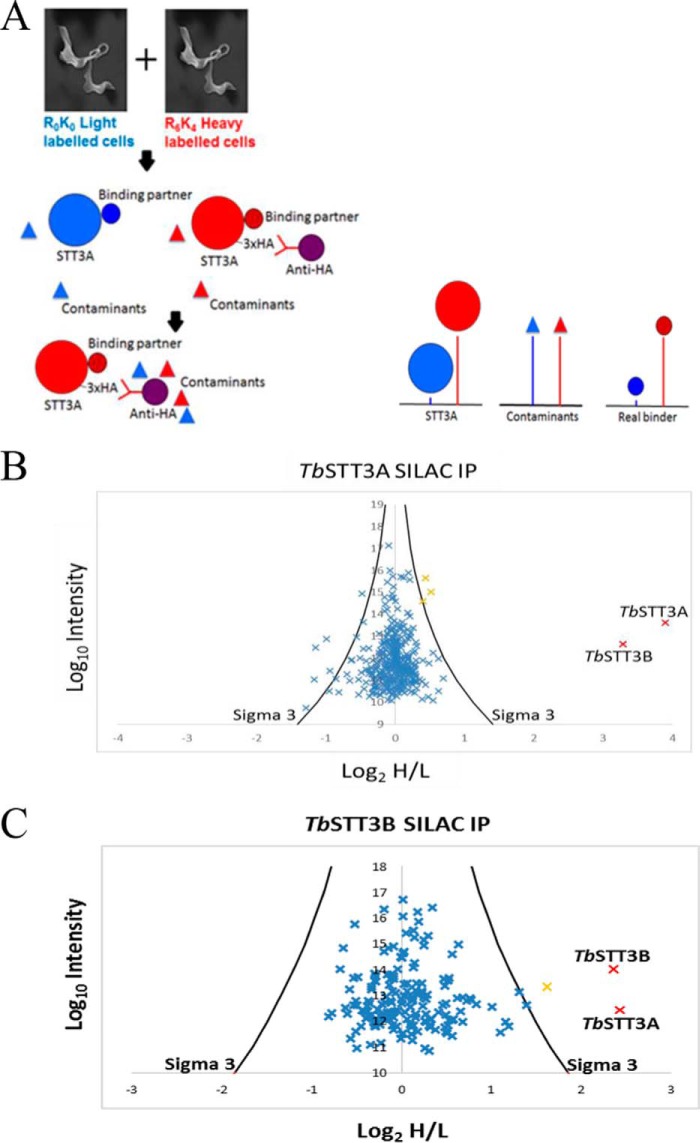
**Overview of the SILAC pull-out experiment and plot of proteomics data.** *A,* overview of the SILAC experiment. Wild-type bloodstream-form cells were grown in light (R0K0) medium, and cells expressing i*n situ*-tagged *Tb*STT3A-HA3 were labeled with heavy (R6K4) medium. The cells were mixed 1:1 and lysed with digitonin, and *Tb*STT3A-HA3 was enriched by affinity selection on anti-HA magnetic beads. Peptides from *Tb*STT3A-HA3 and genuine binding proteins have high heavy/light isotope ratios, whereas those from contaminants will have ratios close to 1:1 (log2 = 0). *B,* results from the *Tb*STT3A SILAC pull-out experiment. The plot shows the log2 of the heavy-to-light isotope ratio (*x* axis) *versus* the log10 value of the intensities of the peptides belonging to each protein that was detected (*y* axis). The *black curves* (marked sigma 3) represent three standard deviations from the mean. Proteins plotted in *orange* have a heavy-to-light ratio above the sigma 3 cut off and are significantly enriched. *Tb*STT3AHA3 (bait) and *Tb*STT3B (both annotated) were shown to be highly enriched and are highlighted in *red. C,* results from the *Tb*STT3B-Myc3 SILAC pull-out experiment. The plot is the same as in *B* except that *in situ*-tagged *Tb*STT3B-Myc3 was used as bait. Again, *Tb*STT3A and *Tb*STT3A (both annotated and highlighted in *red*) were significantly enriched.

Because the results from the blue native gel electrophoresis suggested *Tb*STT3A is present in high molecular weight complexes, we carried out pull-out experiments using SILAC. For this experiment, wild-type and transgenic parasites (expressing *Tb*STT3A-HA_3_) were grown under identical conditions for eight cell divisions, except that the transgenic *Tb*STT3A-HA_3_ cell line was grown in “heavy medium” containing stable isotope-labeled Lys and Arg (R_6_K_4_), whereas the wild-type cells were grown in “light medium” containing unlabeled Lys and Arg (R_0_K_0_). The transgenic *Tb*STT3A-HA_3_ and the wild-type cells were harvested, washed, counted, mixed together in a 1:1 ratio, and lysed in 0.5% digitonin buffer. Anti-HA antibodies and protein G magnetic beads were used to pull-out the *Tb*STT3A-HA_3_–tagged protein and any binding partners, and the bead eluate was processed to tryptic peptides for LC-MS/MS analysis. In this kind of SILAC experiment, *Tb*STT3A-HA_3_ and any proteins specifically associated with it can be distinguished from non-specific contaminant proteins by the isotope ratios of their tryptic peptides. Thus, *Tb*STT3A-HA_3_ and true associated protein peptides will have high heavy/light isotope ratios, whereas contaminant proteins will have approximately equal heavy/light isotope ratios (Fig. 2*A*). The data set from the experiment was used to search a *T. brucei* predicted protein database using MaxQuant software. Each protein was displayed on a plot of the log_10_ value of the intensities of the unique peptides of that protein (*y* axis) and the log_2_ value of the heavy to light isotope ratios of the same peptides (*x* axis) (Fig. 2*B*). The *Tb*STT3A-HA_3_ (bait) protein had the highest heavy/light ratio (14:1), closely followed by *Tb*STT3B (10:1). Only three other proteins were significantly enriched (*orange crosses,* Fig. 2*B*). However, these were only marginally (1.5-fold) enriched hits that are not known to localize to the ER.

The *Tb*STT3A-HA_3_ cell line was further modified by the *in situ* tagging of the remaining *Tb*STT3B allele, to yield a cell line expressing C-terminally Myc_3_-tagged *Tb*STT3B-Myc_3_. A complementary SILAC experiment, using *in situ* TbSTT3B-Myc_3_-tagged bait and an anti-Myc pull-out, produced similar results to the *Tb*STT3A-HA_3_ pull-out, with *Tb*STT3A being the only obvious binding partner for *Tb*STT3B-Myc_3_ (Fig. 2*C*). In this case, there was one other significant protein hit (*orange cross,* Fig. 2*C*), corresponding to a glucose transporter, but this was different from those seen in Fig. 2*B,* and also unlikely to be an ER component.

Taken together, these data suggest that *Tb*STT3A and *Tb*STT3A form hetero-oligomeric complexes, with no other candidate subunits, although we cannot rule out the presence of low-affinity subunits that might be lost during immunoprecipitation. The data for the SILAC proteomics experiments can be found at ProteomeXchange under entry PXD007236.

### Co-immunoprecipitation of TbSTT3A-HA3 and TbSTT3B-Myc_3_

**Figure 3.**
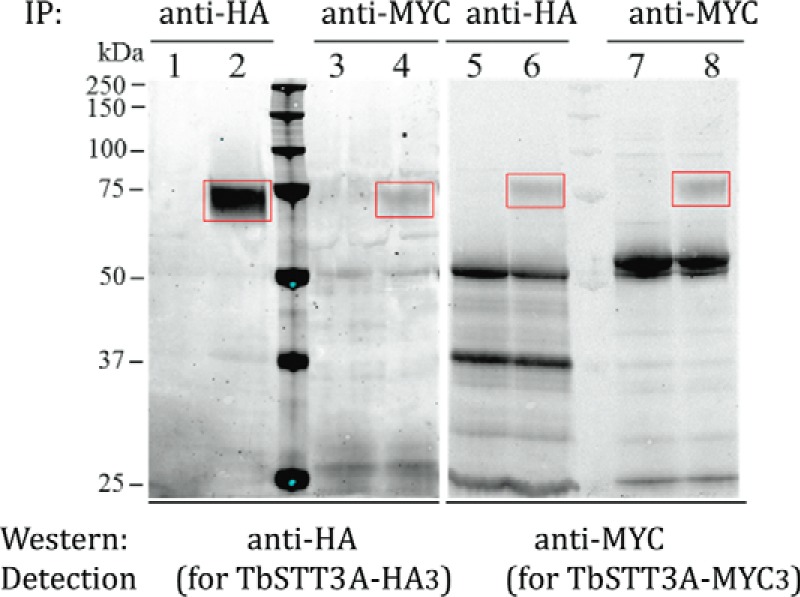
**Co-immunoprecipitation of *Tb*STT3A and *Tb*STT3B.** Digitonin lysates from wild-type cells (*lanes 1, 3, 5,* and *7*) and *Tb*STT3A-HA3 and *Tb*STT3B-Myc3 double *in situ* tagged cells (*lanes 2, 4, 6,* and *8*) were subjected to IP and Western blotted with anti-HA or anti-Myc antibodies, as indicated. The *red rectangles* highlight the bands corresponding to *Tb*STT3AHA3 (*lanes 2* and *4*) and *Tb*STT3B-Myc3 (*lanes 6* and *8*).

The results from the SILAC pull-out experiments suggested that *Tb*STT3A is in a complex with *Tb*STT3B, and to further test this hypothesis, immunoprecipitation (IP) experiments were performed. Cells from the double-tagged cell line were harvested, washed, and lysed in 0.5% digitonin, and *Tb*STT3A-HA_3_ or *Tb*STT3B-Myc_3_ was captured from the lysate using anti-HA or anti-Myc magnetic beads. Subsequently, the tagged proteins were detected by Western blotting using anti-HA and anti-Myc antibodies. Wild-type cell lysates (containing no HA- or Myc-tagged genes) were used as a control. The results from the co-IP experiments are shown in (Fig. 3). As expected, no bands in the region of *Tb*STT3A-HA_3_ or *Tb*STT3B-Myc_3_ were seen in the IPs from the control wild-type lysates (Fig. 3, *lanes 1, 3, 5,* and *7*). Also, as expected, *Tb*STT3A-HA_3_ and *Tb*STT3B-Myc_3_ were detected in the homologous anti-HA IP/anti-HA blot (Fig. 3, *lane 2*) and anti-Myc IP/anti-Myc blot (Fig. 3, *lane 8*). Significantly, *Tb*STT3B-Myc_3_ can be seen to co-IP with *Tb*STT3A-HA_3_ (Fig. 3, *lane 6*), and *Tb*STT3A-HA_3_ can be seen to co-IP with *Tb*STT3B-Myc_3_ (Fig. 3, *lane 4*), confirming their physical association predicted by the SILAC experiment.

In these experiments, the anti-HA IP/anti-HA Western blot signal for *Tb*STT3A-HA_3_ is much stronger than the anti-Myc IP/anti-Myc Western blot signal for *Tb*STT3B-Myc_3_. Whereas some of this difference may be due to relative antibody affinities, it is also consistent with the higher expression of *Tb*STT3A in wild-type bloodstream-form trypanosomes at both the mRNA and protein levels (28, 35). The co-IP data suggest that a significant proportion of the total *Tb*STT3B-Myc_3_ appears in the *Tb*STT3A-HA_3_ IP (Fig. 3, compare *lanes 6* and *8*), whereas only a minority of *Tb*STT3A-HA_3_ appears in the *Tb*STT3B-Myc_3_ IP (Fig. 3, compare *lanes 2* and *4*). These data suggest that there may be some high molecular weight complexes made exclusively, or almost exclusively, of *Tb*STT3A, whereas all, or most, of the *Tb*STT3B is present in complexes containing *Tb*STT3A_._

### Probing peptide acceptor substrate specificities of TbSTT3A and TbSTT3A using a reporter glycoprotein expression system

*Tb*STT3A is responsible for co-translational transfer of biantennary Man_5_GlcNAc_2_ predominantly to *N-*glycosylation sequons containing and/or flanked by acidic amino acids, whereas *Tb*STT3B catalyzes post-translational transfer of triantennary Man_9_GlcNAc_2_ to the remaining sterically accessible sequons (28). To improve our understanding of the acceptor peptide specificity in *T. brucei*, an *in vivo* assay was established using an artificial reporter glycoprotein, based on the *Tb*BiPN system described previously (36). *Tb*BiPN is a non-glycosylated truncated version of *Tb*BiP, retaining its N-terminal signal peptide but lacking its C-terminal ER retention peptide, which enters the ER and is eventually secreted out of the cell via the Golgi apparatus. A pLEW82 expression plasmid (37) was modified to contain the *Tb*BiPN open reading frame fused to a 3′-sequence into which we could insert additional sequences, via AvrII and MfeI restriction sites, immediately upstream of a C-terminal HA_3_ epitope tag. This construct was used to introduce sequences encoding a single reporter *N-*glycosylation sequon, flanked by five amino acid residues on each side. These TbBiP*N-*(*XXXXX*N*X*T*XXXXX*)-HA_3_ constructs were transformed into bloodstream-form trypanosomes to express the reporter glycoprotein.

We first validated the *in vivo* reporter assay by introducing TEGLLNATDEIAL and TILKSNYTAEPVR into the *Tb*BiPN construct and expressing them in *T. brucei.* The former sequence (with a pI of 3.42) is found in VSG MITat1.8 and is known, in that context, to receive exclusively biantennary Man_5_GlcNAc_2_ from *Tb*STT3A (38), whereas the latter (with a pI of 8.3) is found in the ESAG6 subunit of the transferrin receptor and is known not to be recognized and modified by *Tb*STT3A, and therefore, it receives exclusively triantennary Man_9_GlcNAc_2_ from *Tb*STT3B (5). Aliquots of trypanosome lysates expressing these constructs were treated with and without Endo H or PNGaseF, followed by SDS-polyacrylamide gel and Western blotting with anti-HA antibodies. The endoglycosidase Endo H can only digest triantennary Man_9_GlcNAc_2_ glycans transferred by *Tb*STT3B, whereas PNGaseF can digest both Man_9_GlcNAc_2_ and biantennary Man_5_GlcNAc_2_ transferred by *Tb*STT3A. Thus, distinct digestion patterns, depending on what type(s) of glycan(s) are bound to the sequon asparagine, can be visualized by Western blotting. The *in vivo* reporter assay faithfully recapitulated the experimental data for the VSG MITat1.8 and ESAG6 glycosylation sites, with TEGLLNATDEIAL- and TILKSNYTAEPVR-containing *Tb*BipN glycoproteins occupied predominantly by Endo H-resistant and Endo H-sensitive glycans, respectively (supplemental Fig. S2).

**Figure 4.**
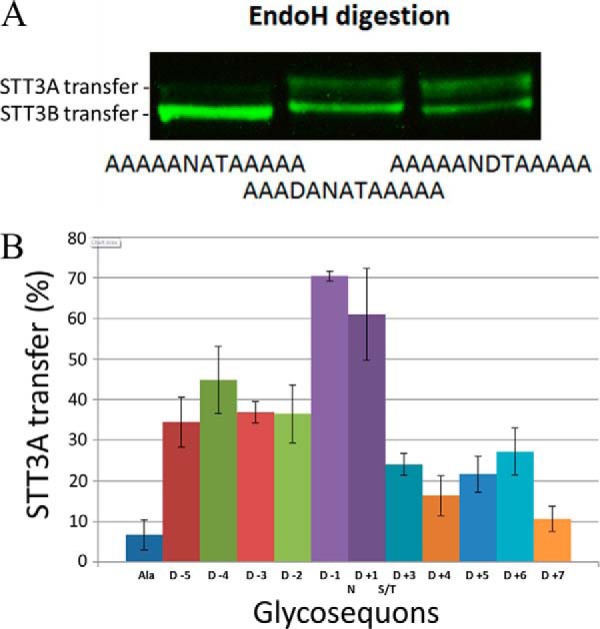
***In vivo* assay of TbSTT3A substrate specificity.** *A,* constructs described in Table 1 were expressed in bloodstream-form trypanosomes, and the resulting TbBiP*N-*(*XXXXX*N*X*T*XXXXX*)-HA_3_ reporter glycoproteins were visualized in cell lysates by SDS-PAGE and anti-HA Western blotting. Representative examples are shown for the sequences indicated. The proportions of the anti-HA signals that were sensitive (*lower bands*) and resistant (*upper bands*) to Endo H were quantified and are reported in Table 1. *B,* summary of the quantitative data from Table 1 showing the influence of replacing single neutral Ala residues with an acidic Asp residue at each possible position.

Next, we investigated how each position flanking and within the sequon affects *Tb*STT3A recognition and transfer. First, the neutral sequence AAAAANATAAAAA (pI 6.01) was introduced into the reporter glycoprotein. For this construct, the majority (about 93% as measured by quantitative Licor imaging of the upper and lower bands of the Endo H digests) of the anti-HA binding signal was sensitive to Endo H (Fig. 4*A*, *1st lane*; Table 1). One aspartic acid was then introduced in all 11 possible positions, yielding peptides with the same pI value (3.10), and the proportion of the reporter glycoprotein processed by *Tb*STT3A (and therefore resistant to Endo H) was measured (Fig. 4*A*, *2nd* and *3rd lanes*; Table 1). The quantitative data, derived from two technical replicates of three biological replicates (Table 1), are summarized in Fig. 4*B*. From these data, it can be seen that the aspartic acid scan across the different positions leads to variation in recognition and glycan transfer by *TbSTT3A*, with the two positions immediately flanking the Asn residue apparently having the greatest influence and with residues N-terminal to the glycosylation sequon having greater influence to those C-terminal to the sequon.

**Table 1:** **Effect of aspartic acid on glycosite recognition by *Tb*STT3A**

Name	Sequence	pI	% EndoH-resistant (% STT3A transfer)*^a^*
Alanine control (Ala)	AAAAANATAAAAA	6.01	6.8 ± 3.7
D −5	**D**AAAANATAAAAA	3.10	34.4 ± 6.1
D −4	A**D**AAANATAAAAA	3.10	44.8 ± 8.9
D −3	AA**D**AANATAAAAA	3.10	36.9 ± 2.6
D −2	AAA**D**ANATAAAAA	3.10	36.5 ± 7.2
D −1	AAAA**D**NATAAAAA	3.10	70.5 ± 1.1
D +1	AAAAAN**D**TAAAAA	3.10	61.0 ± 11.3
D +3	AAAAANAT**D**AAAA	3.10	24.0 ± 2.7
D +4	AAAAANATA**D**AAA	3.10	16.4 ± 4.8
D +5	AAAAANATAA**D**AA	3.10	21.6 ± 4.4
D +6	AAAAANATAAA**D**A	3.10	27.2 ± 5.8
D +7	AAAAANATAAAA**D**	3.10	10.7 ± 3.1

*^a^* The mean and standard deviations of the mean figures are based on *n* = 8 from two technical replicates of three biological replicates.

### Probing endogenous peptide acceptor substrate specificities of TbSTT3A and TbSTT3B by glycoproteomics

The glycoproteomics data from Ref. 28 were reprocessed and significantly augmented by combining them with data derived from the experiments outlined under “Experimental procedures.” These experiments assess whether the endogenous trypanosome *N-*glycosylation sites are occupied by Endo H-sensitive oligomannose glycans (originating from the action of *Tb*STT3B) or by Endo H-resistant paucimannose and/or complex glycans (originating from the action of *Tb*STT3A).

**Figure 5.**
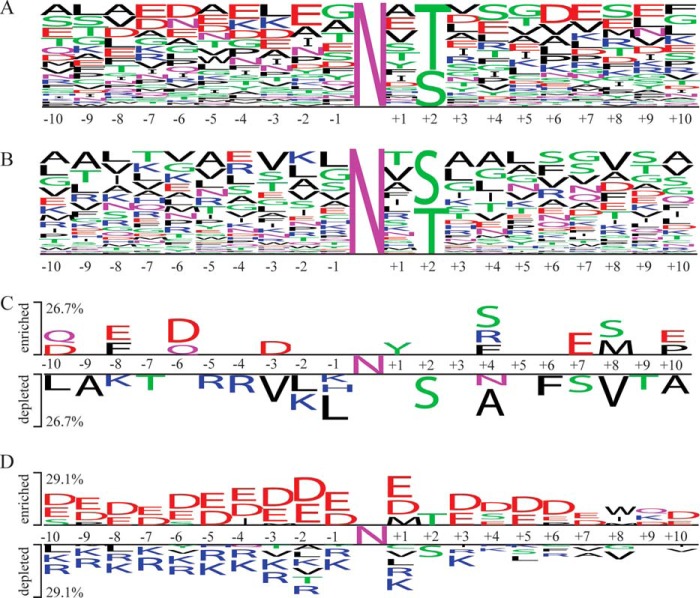
**Glycoproteomic data logos and predictor.** The amino acid frequencies of the preferentially deamidated (*A*) or preferentially HexNAc-modified peptides (*B*) identified by mass spectrometry are visualized with the WebLogo web service. The Two Sample logo web service was used to visualize the amino acids enriched (*upper part*) or depleted (*lower part*) in the sequences of the preferentially deamidated peptides identified by mass spectrometry (*C*) or predicted by the machine learning algorithm (*D*) by using the preferentially HexNAc-modified peptides as negative sample.

Parasites were osmotically lysed to release >90% of the VSG coat as soluble form VSG, through the action of the endogenous GPI-specific phospholipase C (39, 40). The recovered cell ghosts, containing the majority of the non-VSG cellular glycoproteins, were solubilized, denatured, and *S*-alkylated. This preparation was then processed in two ways. In one approach, the intact glycoproteins were first affinity-purified using immobilized ricin (RCA_120_) and concanavalin-A (ConA) lectins. The enriched glycoproteins were then sequentially digested with Endo H and PNGaseF (the latter in the presence of H_2_[^18^O]), and digested with Lys-C and trypsin. In the other approach the denatured and *S*-alkylated proteins were first digested with Lys-C and trypsin, and the glycopeptides were trapped with ricin (RCA_120_) and ConA and subsequently digested with Endo H and PNGaseF (the latter in the presence of H_2_[^18^O]). In both cases, the resulting peptides were analyzed by LC-MS/MS, and the data were used to search the *T. brucei* predicted protein database allowing for the possible presence of Asn-*N-*GlcNAc residues, the product of Endo H cleavage, and/or for the conversion of Asn residues into [^18^O]Asp residues, the product of PNGaseF cleavage. These data and reprocessed data from Izquierdo *et al.* (28), are shown in supplemental Table S1. Peptides containing Asn-*N-*GlcNAc and/or [^18^O]Asp within an Asn-Xaa-Ser/Thr sequon that were detected ≥3 times were assigned as being predominantly *Tb*STT3A or *Tb*STT3B substrates when the proportion of the [^18^O]Asp feature was ≥0.8 and ≤0.4, respectively (supplemental Table S2). We then analyzed the amino acid frequencies immediately adjacent to *N-*glycosylation sequons of the assigned *Tb*TT3A and *Tb*TT3B substrates using WebLogo (41). The enrichment of negatively charged residues is the most striking feature of the *Tb*STT3A substrates (Fig. 5*A*) along with a bias toward Thr over Ser in the sequon +2 position. Conversely, the *Tb*STT3B substrates are relatively enriched for positively charged residues upstream of the sequon but show no preference for Thr over Ser in the +2 position (Fig. 5*B*). The data were also analyzed by the two-sample logo visualization method (42), which compares two input peptide sequence lists against each other, highlighting features that predominate in each. This suggests enrichment for negatively charged amino acids, especially at positions −6, −3, and +7 for the *Tb*STT3A substrates and a preference for hydrophobic and positively charge amino acids in the −1 to −5 positions of the TbSTT3B substrates (Fig. 5*C*). The reprocessed data of Ref. 19 can be found at ProteomeXchange under entry PXD007237 and the new glycoproteomics data under PXD007238.

### Building a glycosylation site predictor for T. brucei using machine learning

Machine learning has been successfully used in biological research to infer the peptide recognition specificities of, for example, protein kinases, phosphatases, and Src homology 2 domains (43). The first step of machine learning consists of transforming peptide sequences into biochemical features such as charge, hydrophobicity, and relative positions. These features, organized in a machine-readable template, are then evaluated by artificial intelligence algorithms to highlight which amino acid properties of a peptide sequence are the most important in determining substrate recognition. We therefore decided to apply a machine learning approach to further leverage our glycoproteomics and glycoprotein reporter data and to build an ensemble of prediction algorithms to assign putative *N-*glycosylation sites in the predicted *T. brucei* proteome. This ensemble algorithm (Voting Classifier) averages the outputs of a Random Forest classifier (RF), an Extra Tree Classifier (ETC), and a Support Vector Machine (SVM) classifier to predict which putative *N-*glycosylation sites will more likely be modified by *Tb*STT3A or *Tb*STT3B. The RF, ETC, and SVM classifiers all weigh the features derived from the upstream (N-terminal) side of the glycosylation sequon more than the features extracted from the downstream (C-terminal) side (supplemental Fig. S3, *A–C*). Moreover, the RF, ETC, and SVM classifiers all preferentially use the cumulative negative charge upstream of the glycosylated asparagine, and the hydrophobicity of the peptide upstream and downstream, to discriminate between *Tb*STT3A and *Tb*STT3B substrates (supplemental Fig. S4). We could detect a core of five important features shared by the three classifiers, namely the charge at pH 7.3 and the isoelectric point for the sequon ±10 amino acid residues, the cumulative charge upstream from the modified asparagine with a window of 13 and 16 amino acids, and the bonus score derived from the aspartic acid scanning experiment described in Fig. 4, see under “Experimental procedures.” A list of putative *N*-glycosylation sites and the Voting Classifier predictions are shown in supplemental Table S3. We performed a two-sample logo visualization on the output (Fig. 5*D*). As expected, the trends in this plot are similar to those generated from the glycoproteomics data alone (Fig. 5*C*).

### Molecular modeling

**Figure 6.**
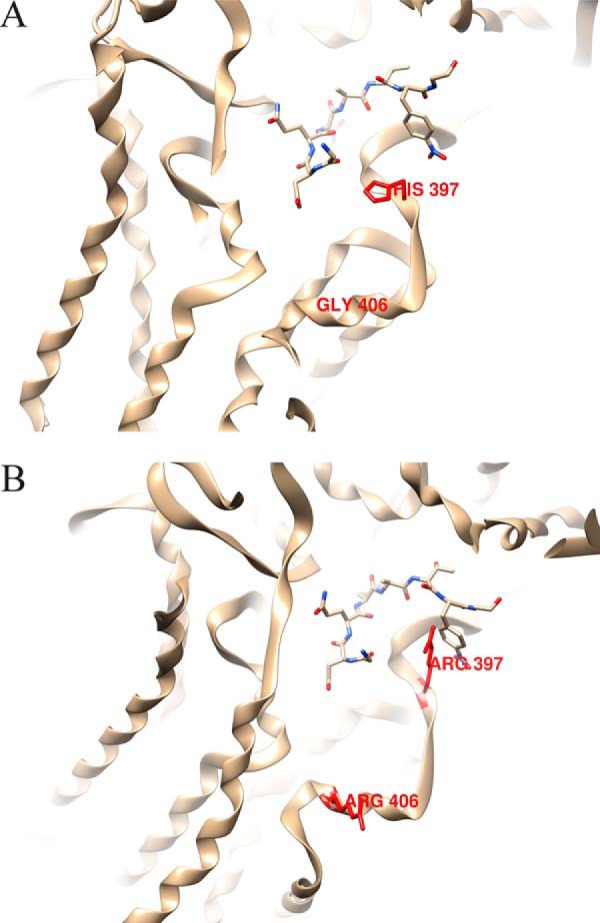
**Molecular models of the active sites of *Tb*STT3A and *Tb*STT3B.** *A* and *B*, molecular models of the predicted active sites of *Tb*STT3A (*A*) and *Tb*STT3B (*B*) with an acceptor peptide (GDQNAT) based on the crystal structure of *C. lari* PglB (46). Of note are the residues in *red*: Arg-397 in *Tb*STT3A (His-397 in *Tb*STT3B) and Arg-406 in *Tb*STT3A (Gly-406 in *Tb*STT3B), where the guanidinium cations of the Arg side chains could interact with acidic residues at or close to the acceptor peptide sequon.

Although *Tb*STT3C (Tb927.5.910) has not been studied in this paper because it is not expressed at detectable levels in bloodstream-form *T. brucei*, data from its heterologous expression in yeast suggest that its peptide acceptor specificity is much more similar to *Tb*STT3A than *Tb*STT3B (28, 44). To try to rationalize the preferences of *Tb*STT3A and *Tb*STT3C for acceptor sequons containing and/or flanked by negatively charged amino acid residues, we built molecular models of *Tb*STT3A, *Tb*STT3B, and *Tb*STT3C using Phyre2 (45) based on the *Campylobacter lari* PglB structure (46). The predicted models were aligned with the PglB structure using PDBeFold (47), and the binding pockets of *Tb*STT3A and *Tb*STT3B were visualized with Chimera (48). Next, we looked for basic amino acid residues that were conserved in *Tb*STT3A and *Tb*STT3C that were different in *Tb*STT3B (supplemental Fig. S6). Of these, the active-site proximal residue 397 is particularly interesting as it contains a His residue in *Tb*STT3B but an Arg residue in *Tb*STT3A and *Tb*STT3C. Arginine has a flexible and strongly basic guanidinium cation side chain that could conceivably interact with acidic amino acid residues at or close to the N*X*(S/T) glycosylation sequon in the active site (Fig. 6). Position 406 contains an Arg residue in *Tb*STT3A and *Tb*STT3C (in place of a neutral Gly residues in *Tb*STT3B) that could also conceivably interact with sequon-adjacent anionic residues in the acceptor peptide (Fig. 6). Such ionic interactions between the acceptor peptide and the enzyme surface might increase the efficiency of substrate recognition and glycosylation of sequons containing and/or flanked by acidic amino acids.

## Discussion

Although the *T. brucei* genome does not encode for any identifiable OST subunits, other than three intact and one truncated STT3, we decided to investigate whether there might be novel non-canonical *T. brucei* OST subunits. Precedents for kinetoplastid-specific subunits of otherwise conserved cellular machineries include clathrin-associating proteins and endocytic components (49, 50), exocyst components (50), nuclear pore complex and nuclear lamina components (51), and subunits of the GPI transamidase (52). Blue native gel electrophoresis of gently solubilized epitope-tagged endogenous *Tb*STT3A showed that it is present in high-molecular-weight complexes, but quantitative SILAC proteomics of tagged *Tb*STT3A and *Tb*STT3B pull-outs showed that although these are mutual binding partners (confirmed by co-immunoprecipitation), no other subunits could be found by these methods. The lack of non-canonical or canonical *T. brucei* OST subunits (other than the STT3 catalytic subunits) is consistent with the ease with which *T. brucei* and other kinetoplastid STT3s can be functionally expressed in other eukaryotes, like *S. cerevisiae* and *Pichia pastoris* (29, 31, 32, 53). Nevertheless, the blue native gel and co-immunoprecipitation experiments show that, at least in the native environment of a bloodstream-form trypanosome, *Tb*STT3A associates with itself and with the less-abundant *Tb*STT3B to form complexes with apparent molecular masses between 600 kDa and 1.2 MDa. The nature of these complexes remains to be determined but has implications for how and whether *Tb*STT3s associate with the parasite translocon complex and how they access nascent glycoprotein sequons during and/or following protein translocation. The dimer/oligomer nature of yeast OST subunits, including Stt3, has been previously described (54).

To probe *Tb*STT3 peptide acceptor substrate specificities, we developed an artificial glycoprotein reporter system, based on a truncated version of *Tb*BiP (36) fused to a single glycosylation sequon flanked by five variable residues on either side. Constructs were expressed in bloodstream-form *T. brucei,* and their products were assayed for the relative proportions of *N*-glycosylation by *Tb*STT3A and *Tb*STT3B. With this, we were able to recapitulate the preferential *N*-glycosylations of native peptide acceptor sequences. We then applied the system to analyze the *N*-glycosylation of an artificial 13-mer sequence (AAAAANATAAAAA) into which we sequentially introduced a single Asp residue in all 11 possible Ala sites. The data clearly confirmed that the presence of an acidic amino acid proximal to the sequon significantly increased its *N*-glycosylation by *Tb*STT3A, with the −1 and +1 positions relative to the *N*-glycosylated Asn residue having the greatest effect and the positions N-terminal to the sequon having a greater effect than those C-terminal to the sequon.

We then created a richer glycoproteomics dataset than we previously reported (28) by combining two alternative approaches: (i) glycoprotein enrichment by lectin affinity chromatography, followed by trypsin digestion and sequential Endo H and PNGaseF digestion; and (ii) tryptic glycopeptide enrichment by lectin affinity chromatography, followed by sequential Endo H and PNGaseF digestion. In both cases, the PNGaseF digestion step was performed in H_2_[^18^O] to distinguish between PNGaseF-mediated Asn deamidation and non-enzymatic deamidation during sample preparation and handling. These data were combined with reprocessed raw data from Ref. 28 to provide quantitative data on *Tb*STT3A and *Tb*STT3B *N*-glycosylation of 141 unique *N*-glycosylation sites. Logo plots confirmed the enrichment of acidic amino acid residues (Asp and Glu) surrounding *Tb*STT3A *N*-glycosylated sequons, the general depletion of hydrophobic residues, and the selective depletion of basic residues (Arg and Lys) N-terminal to the sequon.

We also used hypothesis-free machine-learning techniques to identify features that predispose sequons to be preferentially modified by *Tb*STT3A or *Tb*STT3B, and finally, we combined these features and parameters derived from the experimental reporter glycoprotein data to develop a Voting Classifier prediction algorithm. This predictor was then applied to all the putative *N*-glycosylation sequons in the *T. brucei* proteome to predict those sites preferentially modified by *Tb*STT3A (leading to paucimannose and/or complex *N*-glycan occupancy) or *Tb*STT3B (leading to oligomannose *N*-glycan occupancy) in bloodstream-form trypanosomes. Two-sample logo plot analysis of the output (a total of 1291 predicted occupied *N*-glycosylation sites) largely echoes the experimental glycoproteomic and reporter glycoprotein data and implies that the *Tb*STT3A *N*-glycosylation sites are enriched for acidic residues and depleted of basic and hydrophobic residues, with the effects of these features more profound to the N-terminal side and within the sequon than to the C-terminal side of the sequon.

It is important to note that available pulse-chase data (21, 55) suggest that *Tb*STT3A modifies VSG glycoproteins co-translationally, whereas *Tb*STT3B can act post-translationally (25), and that *Tb*STT3A and *Tb*STT3B knockdown data suggest that *Tb*STT3B is able to modify *Tb*STT3A sites, but not vice versa (28). Thus, all or most of the aforementioned *Tb*STT3A *versus Tb*STT3B sequon selectivity features are dictated by the peptide/sequon acceptor specificity of *Tb*STT3A and not *Tb*STT3B, which appears to be able to utilize sequons in almost any amino acid sequence context. This property of *Tb*STT3B could be particularly useful from a biotechnological point of view to boost the efficient *N*-glycosylation of recombinant glycoproteins in eukaryotic expression systems. It also nicely explains why trypanosomes can transition from expressing a rich mixture of oligomannose *and* paucimannose/complex *N*-glycans in the bloodstream-form parasite (16, 18, 19) to predominantly oligomannose *N*-glycans in the procyclic form of the parasite (12, 20) by simply down-regulating the expression of *Tb*STT3A, as observed at both the mRNA (28, 56) and protein levels (35, 57, 58).

The results reported here and in Ref. 44 are consistent with the mechanisms of resistance in *T. brucei* to certain toxic lectins and carbohydrate-binding small molecules reported in an interesting series of studies (59–61). These workers demonstrated that the parasites could escape the effects of these trypanocidal agents, all of which bind principally to oligomannose *N*-glycans, by either switching to the expression of a VSG type that naturally does not carry oligomannose *N*-glycans or by suppressing the expression of *Tb*STT3B. In this way, the parasites effectively exchange oligomannose for puacimannose and complex *N-*glycans that are poor ligands for the trypanocides.

Interestingly, *Tb*STT3A and *Tb*STT3B are found in tandem array in the trypanosome genome, together with a preceding truncated *Tb*STT3 pseudogene and followed by a full-length *Tb*STT3C gene that is more similar to *Tb*STT3B than to *Tb*STT3A. However, *Tb*STT3C is not significantly expressed in bloodstream-form or procyclic form of the parasite (28, 35, 56–58). Nevertheless, transgenic expression of *Tb*STT3C in *S. cerevisiae* clearly shows that it is a functional OST with a similar preference for sequons flanked by acidic amino acids to *Tb*STT3A but an LLO donor specificity like *Tb*STT3B (28, 44). Amino acid sequence alignment of *Tb*STT3A, -B, and -C and molecular model building, based on the *C. lari* PglB structure (46) was performed. The models suggest that the presence of a large, flexible and highly positively charged Arg residue side chain (Arg-397) very close to the active site of the enzyme in *Tb*STT3A and *Tb*STT3C, compared with a His residue in *Tb*STT3B, may play a role in the selectivity of *Tb*STT3A and *Tb*STT3C for sequons containing and flanked by acidic Asp and Glu residues. *Tb*STT3A and *Tb*STT3C also contain Arg residues in place of neutral Gln-567 and Gly-406 residues in *Tb*STT3B, locations close enough to the active site to potentially interact with sequon-flanking anionic residues. The accompanying paper (44) elegantly addresses the issues of peptide acceptor and LLO donor specificities of all three *Tb*STT3s by heterologous expression of each and chimeras thereof in various yeast mutants. The accompanying paper concludes that the region containing Arg-397 and Arg-406 in *Tb*STT3A and *Tb*STT3C controls peptide acceptor specificity, and this is consistent with our suggestions from molecular modeling.

There are similarities and differences between the multisubunit mammalian STT3A- and STT3B-based OSTs and the single subunit *Tb*STT3A and *Tb*STT3B OSTs of *T. brucei*. (i) In both, the STT3A OSTs operate co-translationally and get the first option to glycosylate a given sequon, whereas the STT3B OSTs can operate post-translationally on what is left (25, 62). (ii) In both, the OSTs show differences in peptide acceptor substrate specificity. However, in *T. brucei* this is controlled by the physicochemical properties of the amino acids surrounding the acceptor sequon, whereas in mammalian cells this is controlled by the position of the sequon relative to the C terminus of the protein (63) or proximity to the signal peptide–cleaved N terminus of the protein and to cysteine residues (23, 63, 64). The latter appears to relate to the presence of the mutually redundant MagT1 or TUSC3 thioredoxin-like oxidoreductase subunits (equivalent to the yeast Ost3 and Ost6 subunits) in the STT3B OST that may form mixed disulfides with the sequon-proximal cysteine residues and thus increase residence time with the STT3B OST (64, 65). A role for oxidoreductase activities of Ost3 and Ost6 in yeast OST acceptor site specificity was also previously shown (66). In this regard, it is worth noting that *Tb*STT3B and *Tb*STT3C contain a C*X*C motif (absent in *Tb*STT3A) that is predicted from the *C. lari* OST structure to be proximal to the acceptor peptide (46). Such C*X*C sequences can have a disulfide isomerase activity (67) that might conceivably increase the acceptor substrate range of *Tb*STT3B and *Tb*STT3C. (iii) Whereas both STT3A and STT3B OSTs prefer the mature Glc_3_Man_9_GlcNAc_2_-PP-dolichol LLO donor, the *T. brucei* OSTs have distinct LLO donor specificities such that the presence of the ALG12-dependent c-branch of the conventional (but glucose-free) triantennary Man_9_GlcNAc_2_-PP-dolichol LLO is required by *Tb*STT3B but not tolerated by *Tb*STT3A (27, 44). An important consequence of this differential LLO specificity is that, because Golgi mannosidase II activity is also absent in *T. brucei*, *N*-glycans derived from *Tb*STT3B glycosylation cannot be processed to paucimannose or complex structures, which must instead be derived exclusively from *Tb*STT3A glycosylation.

In summary, the two simultaneously operating acceptor substrate-specific and donor substrate-specific *N*-glycosylation systems of bloodstream-form *T. brucei* have been further characterized in this paper. Whereas no canonical OST subunits, other than catalytic STT3 subunits, could be found in the parasite genome, we can now confirm that there are no non-canonical subunits either. Instead, *Tb*STT3A and *Tb*STT3B appear to form multimeric high-molecular-weight complexes containing either *Tb*STT3A alone or *Tb*STT3A and *Tb*STT3B. Further insights into the peptide acceptor specificity of *Tb*STT3A have been provided, and an algorithm has been generated to predict, proteome-wide, which OST will likely operate on which putative *N*-glycosylation site. Taken together with the unusual specificities of *T. brucei* UGGT, GnTI, and GnTII enzymes described in the Introduction (19, 33, 34), and the apparent absence of a regulated ER unfolded protein response (19, 68), we may conclude that protein *N*-glycosylation and downstream processing in this divergent eukaryote is worthy of note and that its unusual features may provide therapeutic possibilities.

## Experimental procedures

### Cultivation of trypanosomes

Bloodstream-form *T. brucei*, genetically modified to express T7 polymerase, and the tetracycline repressor protein (37) were cultured in HMI-9T medium (69) supplemented with 10% fetal calf serum, 2 mm Glutamax^TM^ (Invitrogen), 56 μm 1-thioglycerol (in place of 2-mercaptoethanol), and 2.5 μg/ml G418 antibiotic at 37 °C in a 5% CO_2_ incubator. Other antibiotics used, as appropriate, were hygromycin (4 μg/ml), puromycin (2.5 μg/ml), phleomycin (0.1 μg/ml), and tetracycline (0.5 μg/ml). SILAC labeling, using dialyzed fetal calf serum, was performed in HMI11-SILAC media, as described previously (35). l-Arginine U-13C6 and l-lysine 4,4,5,5–2H4 (R6K4) were purchased from Cambridge Isotope Labs.

### Generation of genetically modified trypanosomes with in situ tagged TbSTT3A-HA_3_ and TbSTT3B-Myc_3_

The *Tb*STT3A,B,C^−/+^ heterozygote described in Ref. 28 was used for C-terminal HA_3_ *in situ* tagging of the remaining *Tb*STT3A allele using a pMOTagH4 plasmid and C-terminal Myc_3_ *in situ* tagging of the remaining *Tb*STT3B allele using a pMOTag4M4 plasmid (70). For the pMOTagH4 plasmid, the 1328 bp from the C terminus of *Tb*STT3A and the 1057 bp 3′-UTR downstream of the gene ORF were PCR-amplified from genomic DNA using Kod Hot Start polymerase with primers 5′-ataagtatctcgagcaagtttgcttgccccgttcg-3′ and 5′-ataagtaactcgagctc*gctctgaaaatacaggttttc*gacttcgtaatggaaccgcttcgct-3′ and 5′-ataagtatggatccccacatcgtttcaatcgccgc-3′ and 5′-ataagtaaggatccactcacaatcgtgcttacagcc-3′ as forward and reverse primers, respectively. The PCR products were cloned into the plasmid using the XhoI and BamHI (underlined). A tobacco etch virus restriction site was included downstream of the ORF of *Tb*STT3A (italics). The construct was linearized before being transfected into the *Tb*STT3A,B,C^−/+^ heterozygote cell line, and transfected cells were selected by addition of hygromycin. The pMOTag4M4 plasmid was ordered from Genescript. It included 1032 bp from the C terminus of *Tb*STT3B, located upstream of the Myc_3_ epitope in the plasmid. The plasmid also included 835 bp of the 3′ UTR of *Tb*STT3B, which were located downstream of the blasticidin resistance gene in the plasmid. The construct was linearized before being transfected into the *Tb*STT3A/B/C^−/+^ *Tb*STT3A-HA_3_ heterozygote cell line, and transfected cells were selected by addition of blasticidin.

### SDS-PAGE and Western blotting

Reducing SDS-PAGE was run using pre-cast Novex BisTris gels with MOPS running buffer (Invitrogen). Proteins were transferred to nitrocellulose using an iBlot system (Invitrogen) and stained with Ponceau S (Sigma) before being blocked in 50 mm Tris-HCl, 0.15 m NaCl, 0.05% Tween 20, 0.25% BSA, 0.05% sodium, and 2% fish skin gelatin, pH 7.4, for 20 min. The membrane was then incubated for 30 min with primary antibody in a 50-ml Falcon tube followed by washing using a SnapID system (Millipore). Subsequently, the labeled secondary antibody was incubated for 30 min followed by a washing step. The blots were imaged using an ODYSSEY® SA near infrared imager (LI-COR Biosciences). Secondary LI-COR antibodies (IRDye-800CW goat anti-mouse 1:15,000 or IRDye-680RD donkey anti-mouse 1:20,000) were used to bind the primary mouse anti-HA and anti-Myc antibodies.

### Blue native gels and Western blotting

Blue native gel electrophoresis was run using components from the native PAGE kit (Invitrogen). The protocol followed the manufacturer's instructions except that no G-250 was added to the sample buffer and the 1× native PAGE Light Cathode buffer was diluted 1:4 in 1× running buffer to reduce Coomassie interference of the post-blotting LiCor imaging.

### Immunoprecipitation

Cells cultures (100 ml) were grown to log phase (∼2.5 × 10^6^ cells/ml) and lysed for 30 min on ice in 0.5% digitonin, 50 mm Tris-HCl, pH 6.8, 20 mm EDTA plus the protease inhibitors 0.8 mm PMSF, 0.1 mm TLCK, and 1× EDTA-free protease inhibitor mixture (Roche Applied Science). Subsequently, the lysate was centrifuged (4 °C, 11,000 × *g*, 15 min), and the supernatant was moved to a new tube. Protein G magnetic beads, pre-washed in lysis buffer, were added to the lysate (30 min, 4 °C) and captured to absorb non-specific binding components. The lysate was moved to a new tube followed by incubation for 1 h with anti-HA or anti-Myc antibody (1 μg/ml) at 4 °C followed by fresh pre-washed magnetic beads. The beads were captured and washed twice with 1 ml of lysis buffer and once with 10 mm Tris-HCl, pH 6.8, 4 mm EDTA, 0.1% digitonin containing the same protease inhibitors. Proteins were eluted from the beads in 30 μl of reducing SDS-sample buffer with heating (100 °C for 10 min). The eluted proteins were subsequently separated by SDS-PAGE.

### SILAC proteomics

Heavy and light labeled cells were harvested separately (15 min, 800 × *g*, 4 °C) and washed and resuspended in trypanosome dilution buffer (20 mm Na_2_HPO_4_, 2 mm NaH_2_PO_4_, 80 mm NaCl, 5 mm KCl, 20 mm glucose) for cell counting. The cells were mixed 1:1 before undergoing immunoprecipitation, as described above. The eluted proteins in 25 μl of reducing SDS sample buffer were *S*-alkylated with 5 μl of 300 mm iodoacetamide (30 min, dark) and loaded on Novex NUPAGE 4–12% BisTris gel and run at 200 V using MOPS buffer until the proteins had migrated about 2 cm into the gel (visualized by Simply Blue Safe Stain, Thermo Fisher Scientific). The protein-containing region of the gel was excised and subjected to in-gel trypsin digestion, and aliquots of the extracted peptides were analyzed on an LTQ-Orbitrap Velos Pro mass spectrometer coupled with a Dionex Ultimate 3000 RS HPLC system (Thermo Fisher Scientific). The sample peptides were loaded at 5 μl/min onto a trap column (100 μm × 2 cm, PepMap nanoViper C18 column, 5 μm, 100 Å, Thermo Fisher Scientific) equilibrated in 98% buffer A (2% acetonitrile and 0.1% formic acid (v/v)) and 2% buffer B (80% acetonitrile and 0.08% formic acid (v/v)). The trap column was washed for 3 min at the same flow rate and then switched in-line with a Thermo Fisher Scientific resolving C18 column (75 μm × 50 cm, PepMap RSLC C18 column, 2 μm, 100 Å). The peptides were eluted from the column at a constant flow rate of 300 nl/min with a linear gradient from 98% buffer A to 40% buffer B in 128 min and then to 98% buffer B by 130 min. LTQ-Orbitrap Velos Pro was used in data-dependent mode. A scan cycle comprised an MS1 scan (*m/z* range from 335 to 1800) in the Orbitrap (resolution 60,000) followed by 15 sequential data-depandant collision-induced dissociation MS2 scans (the threshold value was set at 5000 and the minimum injection time was set at 200 ms).

### Glycoproteomics

The protein-based approach was based on the methodology described previously (28). Cells were harvested by centrifugation and osmotically lysed at 3.5 × 10^8^ cells/ml for 5 min at 37 °C in the presence of 0.1 μm TLCK, 1 mm benzamidine, 1 mm phenylmethylsulfonyl fluoride (PMSF), 1 μg/ml leupeptin, 1 μg/ml aprotinin, and Phosphatase Inhibitor Mixture II (Calbiochem). Cell ghosts from a total of 3.5 × 10^9^ trypanosomes, enriched for non-VSG cellular glycoproteins, were collected by centrifugation (16,000 × *g*, 15 min, 4 °C) and solubilized in 250 μl of detergent buffer (4% SDS, 0.1 m DTT, 0.1 m Tris-HCl, pH 7.5) using probe sonication for 30 s before and after heating to 85 °C for 20 min. *S*-Alkylation was performed by mixing with 250 μl of 8 m urea, 0.5 m iodoacetamide, 0.1 m Tris-HCl, pH 8.5, for 1 h, room temperature, in the dark. Unreacted iodoacetamide was quenched by the addition of 10 μl of the detergent buffer. After centrifugation (16,000 × *g*, 15 min), the supernatant was transferred to a filtration device with a 30-kDa molecular cutoff (Sartorius), and the majority of the detergent was removed by diafiltration with 8 m urea, 0.1 m Tris-HCl, pH 8.5, followed by lectin-binding buffer (1 mm CaCl_2_, 1 mm MnCl_2_, 150 mm NaCl in 40 mm Tris-HCl, pH 7.4). An aliquot (400 μg of protein) was mixed with 150-μl packed volume of ricin (RCA_120_)-agarose beads (Vector Laboratories) and rotated gently for 2 h. The beads were washed with lectin-binding buffer and eluted with 300 μl of 30 mg/ml lactose and 30 mg/ml galactose (Sigma) in 40 mm Tris-HCl, pH 7.4. After overnight incubation, the supernatant containing the eluted glycoproteins was collected by centrifugation (10,000 × *g* for 10 min). ConA-coupled agarose beads (0.15 ml packed volume, Vector Laboratories) was added to the recovered supernatant from the RCA_120_ pulldown and incubated at 4 °C overnight. The beads were washed with lectin-binding buffer and eluted with 300 μl of 0.5 m methyl-α-d-mannopyranoside (Sigma) in 40 mm Tris-HCl, pH 7.4. After gentle rotation for 2 h, the supernatant containing glycoproteins was recovered by centrifugation. The RCA_120_ and ConA-eluted glycoproteins were mixed, transferred to a 30-kDa filter, the buffer exchanged with 25 mm ammonium acetate, pH 5.5, and subjected to digestion with 180 milliunits of Endo H (Roche Applied Sciences) overnight at 37 °C. The Endo H-released glycans were removed by centrifugation, and the remaining material was exchanged into 40 mm ammonium bicarbonate buffer in H_2_[^18^O] (Sigma), and subsequently digested with 100 units of *N-*glycosidase F (PNGaseF, Roche Applied Sciences) dissolved in H_2_[^18^O]. PNGaseF in the presence of H_2_[^18^O] converts Asn to [^18^O]Asp with a mass increment of 2.9890 Da that can be readily distinguished from spontaneous deamidation (mass increment of 0.9858 Da). After overnight incubation at 37 °C, the PNGaseF-released glycans were removed by centrifugation, and the deglycosylated proteins were diafiltered into 40 mm ammonium bicarbonate and subsequently digested with a mixture of 1:100 (enzyme/substrate) Lys-C and 1:20 trypsin (Roche Applied Sciences) at 37 °C for 48 h. The peptides were collected by centrifugation through the filter, dried in a Speedvac, and desalted using a ZipTip C18 micro-column (10 μl, Merck Millipore) prior to liquid chromatography tandem mass spectrometry (LC-MS/MS).

An alternative (glyco)peptide-based approach was also performed, based on the method of Ref. 71. An aliquot of the denatured and *S*-alkylated sample (400 μg) was first digested on a 10-kDa filter with a mix of Lys-C and trypsin, as described above. The (glyco)peptides were collected by centrifugation through the filter in lectin-binding buffer and were mixed with lectin solution containing a mixture of ConA and RCA_120_ resulting in mixtures of (glyco)peptides and lectins with a mass proportion of 1:2. After gentle rotation for 2 h, the mixtures were transferred to a 30-kDa filter, and the lectin-captured glycopeptides were diafiltered into 25 mm ammonium acetate, pH 5.5, and subjected to Endo H digestion. After overnight incubation at 37 °C, the Endo H-released peptides (Asn *N-*GlcNAc residue) were collected by gentle centrifugation. The peptides containing Endo H-resistant glycans still bound to lectins were diafiltered into 40 mm ammonium bicarbonate buffer in H_2_[^18^O] and digested with PNGaseF overnight at 37 °C. The de-glycosylated peptides were collected by centrifugation, mixed with the Endo H released fraction, dried in a Speedvac, and desalted using ZipTip C18 prior LC-MS/MS, which was performed as described above.

### LC-MS/MS analysis and data processing

For glycoproteomics, the LC was performed on a fully automated Ultimate U3000 Nano LC System (Dionex) fitted with a C18 trap (PepMap nanoViper, Thermo Fisher Scientific) and resolving columns (PepMap RSLC) with inner diameters of 100 and 75 μm and lengths of 2 and 50 cm, respectively. Mobile phases consisted of 0.1% formic acid (Sigma) in 2% acetonitrile (Merck, Darmstadt Germany) for solvent A and 0.08% formic acid in 80% acetonitrile for solvent B. Samples were loaded in solvent A. A linear gradient was set as follows: 0% B for 5 min and then a gradient up to 40% B in 122 min and to 98% B in 10 min. A 20-min wash at 98% B was used to prevent carryover, and a 20-min equilibration with 2% B completed the gradient. The LC system was coupled to an LTQ-Orbitrap Velos mass spectrometer (Thermo Fisher Scientific) equipped with an Easy spray ion source and operated in positive ion mode. The spray voltage was set to 2 kV and the ion transfer tube at 250 °C. The full scans were acquired in a Fourier transform MS mass analyzer that covered an *m/z* range of 335–1800 at a resolution of 60,000. The MS/MS analysis was performed under data-dependent mode to fragment the top 15 precursors using collision-induced dissociation. A normalized collision energy of −35 eV, an isolation width of *m/z* 2.0, an activation *Q* value of 0.250, and a time of 100 ms were used. The raw files were converted to mgf format by MSConvert software from ProteoWizard (proteowizard.sourceforge.net).^4^ The searches were carried out against the *T. brucei* 927 annotated proteins database (version 8.0, downloaded from TriTrypDB (72), www.tritrypdb.org/)^4^ using Mascot software (version 2.4.0, Matrix Science Inc., Boston). The search parameters for Mascot software were set as follows: peptide tolerance, 5 ppm; MS/MS tolerance, 0.5 Da; enzyme, trypsin; one missed cleavage allowed; and fixed carbamidomethyl modifications of cysteines. Oxidation of methionine, *N-*acetylglucosamine modification of Asn, and deamidation of Asn to Asp containing a single ^18^O atom (2.9890 Da mass increase) were used as variable modifications.

### Dataset extraction

We extracted from the Mascot result files all the peptides with an ion score of >20. From this we selected 350 glycosylated sites with a deamidation (Asn to [^18^O]Asp conversion) or Asn–*N-*HexNAc modification embedded in the N[caret]P(ST) consensus. The list was used to count the number of times (≥ 3) that these changes were detected for each peptide. To increase the number of modified peptides we decided to re-process a previously published work in our laboratory (28). This dataset was re-searched with the same Mascot parameters and database used for this publication. This made it possible to include 14 new peptides preferentially HexNAc-modified and to compile a list of 186 peptides used for the next phase of machine learning implemented in Python with the scikit-learn package (73).

The data set reported here and that from Ref. 28 identified 170 common and 180 and 155 unique glycosylation sites, respectively (supplemental Fig. S1*A*). To check the consistency of the two datasets, we selected 92 peptides that were observed ≥ 4 times in both datasets and plotted the frequencies of the Asn–*N-*HexNAc and Asn to [^18^O]Asp modifications. The two datasets had a good correlation (*r*^2^ = 0.83) (supplemental Fig. S1*B*). However, the experimental procedures used to generate the 2009 dataset (*i.e.* without the use of H_2_^18^O) cannot discriminate between the spontaneous non-enzymatic deamidation of asparagine to aspartic acid *versus* the PNGaseF-mediated deamidation produced during the cleavage of the *N-*glycan. From the linear regression, we could deduce that non-enzymatic deamidation contributed a significant amount (about 18%) of the total deamidation seen in the 2009 dataset (supplemental Fig. S1*B*). For this reason, we only used Asn–*N-*HexNAc containing (TbSTT3B substrate) peptides (with a frequency of ≥ 0.6) from this dataset to augment our machine-learning training set.

### Machine learning

The deamidation proportion was computed for each peptide as (DC/DC + HC), where DC is the Deamidation Count (*i.e.* the number of times a peptide with [^18^O]Asp is detected), and HC is the HexNAc Count (*i.e.* the number of times a peptide with Asn–*N*-HexNAc is detected). This score was used to classify each peptide as preferentially deamidated (score >0.8 *n* = 70) or preferentially HexNAc-modified (score <0.3% *n* = 56). This dataset was used to extract a sequence-based feature from 10 amino acids before and after the glycosylated asparagine with the ASAP package in Python (74). We also added some in-house features derived from the knowledge of the *Tb*STT3A recognition and transfer experiment reported in Fig. 4*B* and Table 1. To this end we created “Bonus Features” for each residue position probed in that experiment based on the increased transfer efficiency observed when that site is occupied by an Asp (or, by inference, a Glu residue). We also included a “Bonus All” feature that summed all of the bonus scores for the peptide when it contained more than one Asp and/or Glu residue and a “Bonus Max” feature that selected only the highest bonus score in such cases. Finally, we also created “Bonus Presence D” and “Bonus Presence E” features that simply recorded the presence or absence of Asp or Glu, respectively, at each residue location. We then developed three machine learning algorithms: an RFC, an ETC, and a support vector machine classifier (SVM). The predictors were used to extract the importance of all the features and to rank the features with recursive feature elimination and cross-validated selection of the best number of features (RFECV methodology). The selected features were used to train the three machine learning algorithms (RFC, ETC, and SVM) that were further optimized using a Bayesian global optimization with Gaussian processes (https://github.com/fmfn/BayesianOptimization).^4^ The optimized machine learning algorithms were combined in a voting classifier to produce our final predictor. The ability of the developed classifiers (RFC, ETC, SVM, and Voting Classifier) to discriminate between the deamidated or HexNAc-modified peptides was assessed with the area under the curve of the receiver-operating characteristic curve. The area under the curve score was computed 100 times with a 5-fold cross-validation, using each time a different random split of the original dataset (supplemental Fig. S5).

## Author contributions

M. A. J. F. conceived and coordinated the study. A. J., L. A., and M. A. J. F. designed the experiments. A. J. and M. L. S. G. performed the biochemical and SILAC proteomic experiments. L. A. performed the glycoproteomic experiments. A. J. and M. T. performed the molecular modeling. M. T. performed the bioinformatic analyses and designed the machine-learning experiments.

## Supplementary Material

Supplemental Data

## Footnotes

4 Please note that the JBC is not responsible for the long-term archiving and maintenance of this site or any other third party hosted site.

## Footnotes

3 The abbreviations used are:

GPI: glycosylphosphatidylinositol
OST: oligosaccharyltransferase
VSG: variant surface glycoprotein
ISG: invariant surface glycoprotein
RFC: random forest classifier
ETC: Extra Tree Classifier
SVM: Support Vector Machine
BisTris: 2-[bis(2-hydroxyethyl)amino]-2-(hydroxymethyl)propane-1,3-diol
TLCK: 1-chloro-3-tosylamide-7-amino-2-heptanone
Endo H: endoglycosidase H
PNGaseF: peptide:*N*-glycosidase-F
ER: endoplasmic reticulum
IP: immunoprecipitation
SILAC: stable isotope labeling in cell culture
UGGT: UDP-glucose–glycoprotein glucosyltransferase
LLO: lipid-linked oligosaccharide
ConA: concanavalin-A.
