# Supplementary material for: Single-subunit oligosaccharyltransferases of Trypanosoma brucei display different and predictable peptide acceptor specificities
Source: J Biol Chem. 2017 Sep 19;292(49):20328–41. doi: 10.1074/jbc.M117.810945 (PMC5724017; doi:10.1074/jbc.M117.810945)
Supplement: Supplemental Data [file supp_292_49_20328__index.html]

Single-subunit oligosaccharyltransferases of Trypanosoma brucei display different and predictable peptide acceptor specificities. — Single-subunit oligosaccharyltransferases of Trypanosoma brucei display different and predictable peptide acceptor specificities — N-Glycosylation in Trypanosoma brucei — Supplemental Data 

# Single-subunit oligosaccharyltransferases of *Trypanosoma brucei* display different and predictable peptide acceptor specificities

## Supplemental Data

- Supplementary Figures (.pdf, 3.0 MB) - Supplementary Figures and Legends
- Table S1 (.xlsx, 105 KB) - Table S1
- Table S2 (.xlsx, 41 KB) - Table S2
- Table S3 (.xlsx, 91 KB) - Table S3
