# Supplementary material for: Single-subunit oligosaccharyltransferases of Trypanosoma brucei display different and predictable peptide acceptor specificities
Source: J Biol Chem. 2017 Sep 19;292(49):20328–41. doi: 10.1074/jbc.M117.810945 (PMC5724017; doi:10.1074/jbc.M117.810945)
Supplement: Supplemental Data [file 10.1074_M117.810945_jbc.M117.810945-1.pdf]

Supplementary data for:

Two single-subunit oligosaccharyltransferases of *Trypanosoma brucei* associate with each other and display different and predictable peptide acceptor specificities.

Anders Jinnelov, Liaqat Ali, Michele Tinti and Michael A.J. Ferguson*
Wellcome Centre for Anti-Infectives Research, School of Life Sciences, University of Dundee, Dundee DD1 5EH, U.K.

Figure legends:

Fig. S1. *In situ* tagging of *Tb*STT3A. Panel A: Predicted digestion pattern for Southern blot analysis of *in situ* tagged *Tb*STT3A. The HA_3 tag is highlighted in green, an intergenic region in purple and the *HYG* resistance gene in grey. When probing against *HYG* following PstI digestion, two bands should be observable at 4.6 and >10 kb and these fragments were visible following Southern blotting. Panel B: VSG221 visualised following PNGaseF or EndoH treatment and Coomassie blue staining of the SDS-PAGE gel. Untreated (lane 1), EndoH (lane 2) and PNGaseF (lane 3) treated samples from a control wild-type cell line showing the typical digestion pattern for native VSG221. Lanes 4-6 show untreated (lane 4), EndoH (lane 5) and PNGaseF (lane 6) treated samples from the *in situ* tagged cell line. Since the digestion pattern resembles that from wild-type cells, we conclude that the product of the single C-terminal HA_3 tagged *Tb*STT3A allele was able to transfer EndoH resistant $Man_5GlcNAc_2$ to N263.

Fig. S2. Glycosylation reporter construct and system validation. The pLEW82 plasmid (Wirtz et al., 1999) drives expression of both the BipN fusion protein and the phleomycin resistance gene (*BLE*) via a T7 promotor. The plasmid is tetracycline-inducible and has one tetracycline operator and two T7 terminators. *Tb*BipN (blue) is fused to an NAT *N*-glycosylation sequon flanked by five variable residues (purple) preceded and followed by Ala_3 linker regions (green), AvrII and MfeI restriction sites (pink) and followed by an HA_3 epitope tag (red). When *Tb*BipN is expressed, the protein can carry a biantennary paucimannose or complex *N*-glycans originating from $Man_5GlcNAc_2$ transferred by *Tb*STT3A or a triantennary oligomannose *N*-glycans originating from $Man_9GlcNAc_2$ transferred by *Tb*STT3B or a mixture of both. Experimental examples showing the EndoH- and PNGaseF-sensitivity of the glycosylated TILKSNYTAEPVR site and the EndoH-

resistance and PNGaseF-sensitivity of the glycosylated TEGLLNATDEIAL site are shown at the bottom. These agree with literature descriptions of these sites (Mehlert et al., 2010, 2012).

Fig. S3. Optimisation of peptide length for classifying *Tb*STT3A and *Tb*STT3B catalysed *N*-glycosylation. The receiver operator curve (ROC) area under the curve (AUC) values of the three different machine learning algorithms were used as a proxy to study the relative importance of peptide sequence length surrounding the glycosylated asparagine. The ROC AUC scores (y axes) are plotted against the numbers of amino acids to the right (red), left (purple) and both (blue) used in the training of the (A) Extra Tree classifier, (B) Random Forest classifier and (C) of the Support Vector Machine classifier.

Fig. S4. Selection of key features for machine learning training. The amino acid sequence features used by the Extra Tree classifier (n=19), the Random Forest classifier (n=8) and the Support Vector Machine classifier (n=73) are analysed with a Venn diagram. The figure shows the names of the features that are considered important at least by two classifiers. These are: The figure shows the names of the features that are considered important at least by two classifiers. These are: aa_count:V: the count of valine amino acids in the peptide sequence, acc_charge_left@X: accumulating charge from the left side of the peptide with a window of X amino acids, bonus_all: the sums of all the bonus scores for the peptide, bonus_max: the highest bonus score for the peptide, bonus_presence_D: the presence or absence of Aspartic Acid (D) in the peptide sequence, PH_Charge_X: the charge of the peptide at pH X, PI: isoelectric point value of the peptide, scale_Atch_X_window_Y: Atchely amino acid scale value with a segment of X amino acids and a window of Y amino acids, scale_polarizability_window_17: amino acid polarizability scale value with a window of 17 amino acids.

Fig S5. Predictor Scores. The figure shows box plots of the scores (5-fold cross validation) for the ROC AUC values (y axis) of the individual RF, ETC and SVM machine learning algorithms and the final combined (Voting Classifier) machine learning algorithm. Each score is computed 100 times with a different random split of the original dataset.

Fig. S6. Amino acid sequence alignment of *Tb*STT3A, B and C. Putative transmembrane domains are indicated in grey and positively charged amino acids found in *Tb*STT3A and *Tb*STT3B, but not found in *Tb*STT3B, are highlighted in red boxes.

Table legends:

Supplementary Table S1. Glycoproteomics Data. Glycopeptides identified from the glycoproteomics experiments described in this paper and the reprocessed data of (Izquierdo et al., 2009b).

Supplementary Table S2. Peptide count data used to discriminate TbSTT3A from TbSTT3B substrates. Peptides containing Asn-N-GlcNAc and/or [^{18}O]Asp within NXS/T sequons identified >=3 times were assigned to being predominantly *Tb*STT3A or TbSTT3B substrates when the proportion of [^{18}O]Asp containing forms were >=0.8 and =<0.4, respectively.

Supplementary Table S3. Machine learning predictions. The table reports the Voting Classifier prediction results of the NXS/T (X≠P) sequons in the *T. brucei* proteome for protein with an N-terminal signal peptide. The protein identification numbers (Protein Id), the protein descriptions (Protein Description), and positions (Protein Site) of the sequons are reported along with the peptide sequence window (Predicted Peptide) used for the prediction. Finally, the table reports the score of the Voting Classifier (Predictor Score) with the prediction of the OST enzyme (Predicted Enzyme) that is more likely to modify the NXS/T sequons.

Fig S1

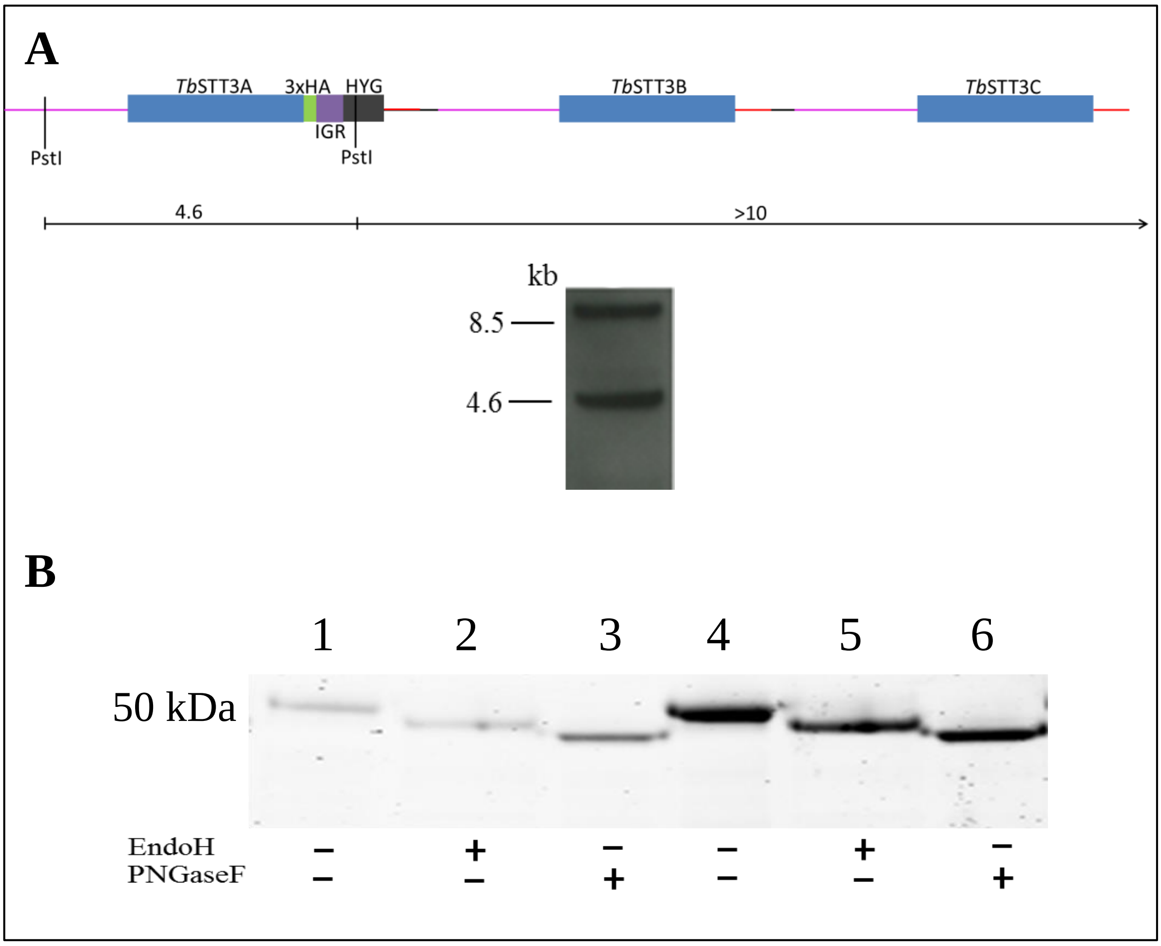
A
TbSTT3A
3xHA
HYG
IGR
PstI
PstI
TbSTT3B
TbSTT3C
4.6
>10
kb
8.5
4.6
B
1
2
3
4
5
6
50 kDa
EndoH
PNGaseF
–
–
+
–
–
+
–
–
+
–
–
+

Fig S2

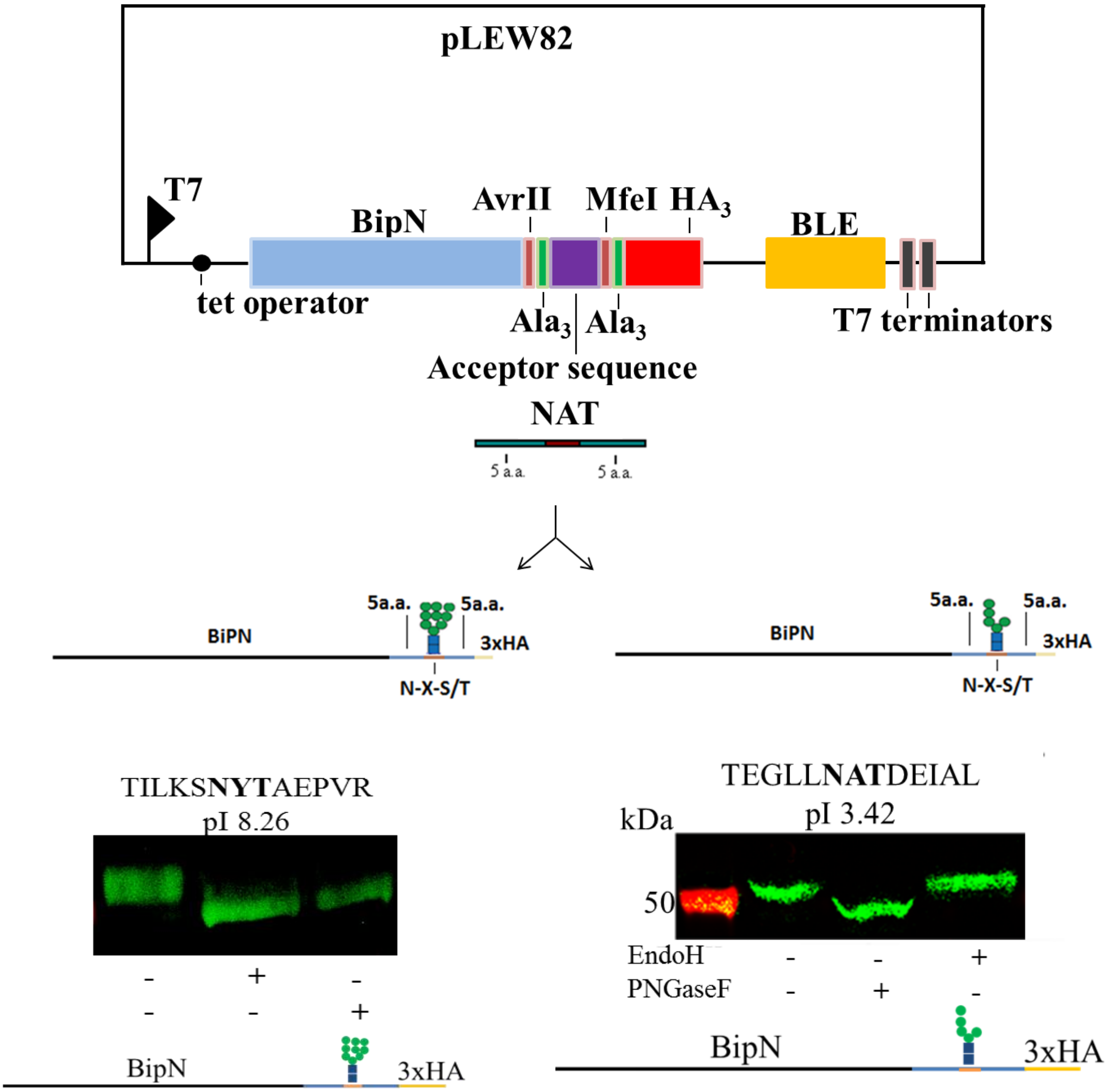
pLEW82
T7
BipN
AvrII
MfeI
HA_3
BLE
tet operator
Ala_3
Ala_3
T7 terminators
Acceptor sequence
NAT
5 a.a.
5 a.a.
BiPN
5a.a.
5a.a.
3xHA
N-X-S/T
BiPN
5a.a.
5a.a.
3xHA
N-X-S/T
TILKSNYTAEPVR
pI 8.26
-
+
-
-
-
+
BipN
3xHA
TEGLLNATDEIAL
pI 3.42
kDa
50
EndoH
-
-
+
PNGaseF
-
+
-
BipN
3xHA

Fig S3

A

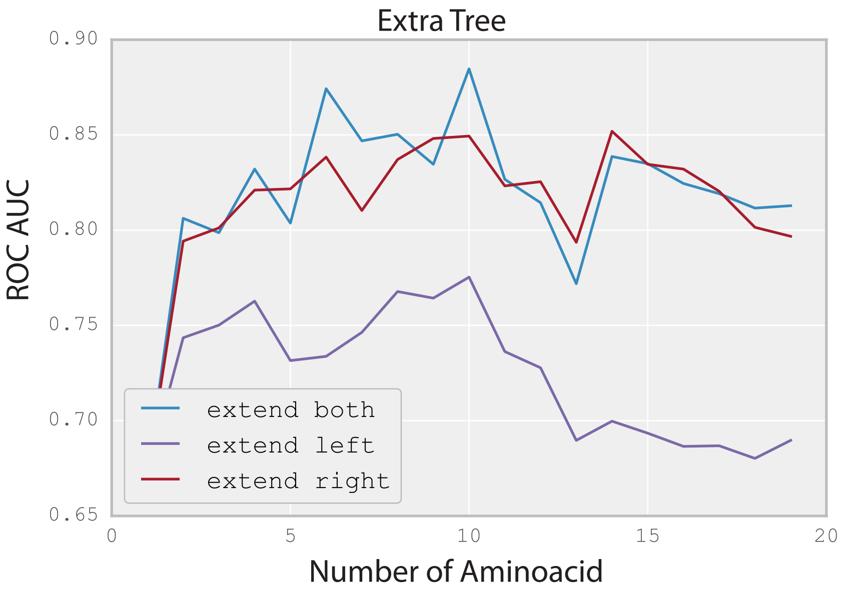
Extra Tree
ROC AUC
0.90
0.85
0.80
0.75
0.70
0.65
0
5
10
15
20
Number of Aminoacid
extend both
extend left
extend right

B

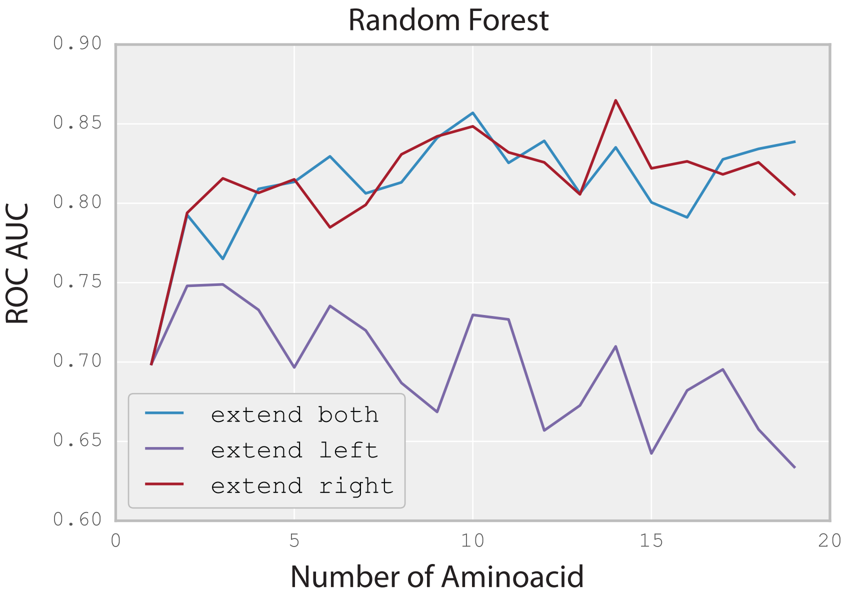
Random Forest
ROC AUC
0.90
0.85
0.80
0.75
0.70
0.65
0.60
0
5
10
15
20
Number of Aminoacid
extend both
extend left
extend right

C

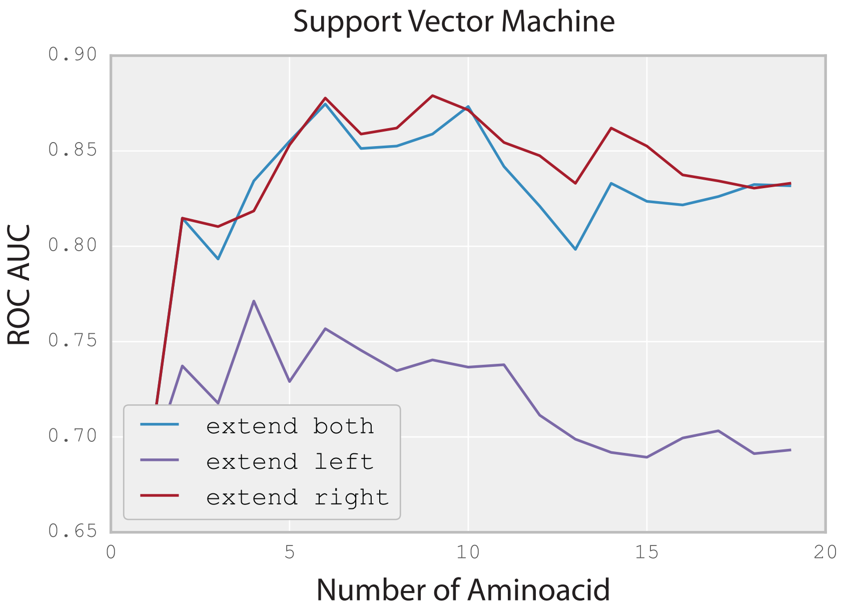
Support Vector Machine
ROC AUC
0.90
0.85
0.80
0.75
0.70
0.65
0
5
10
15
20
Number of Aminoacid
extend both
extend left
extend right

Fig S4

Important features after optimization

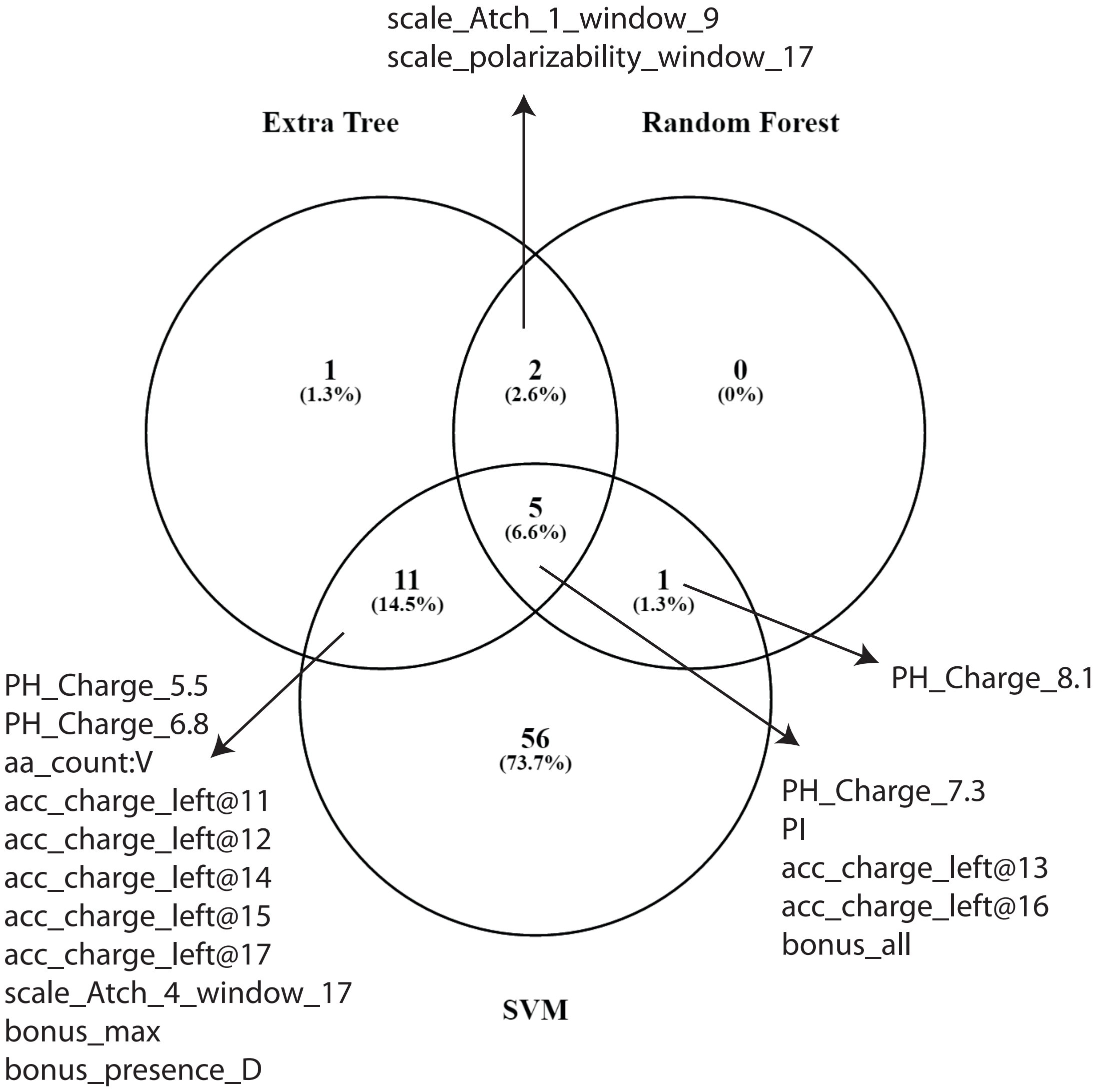

Fig S5

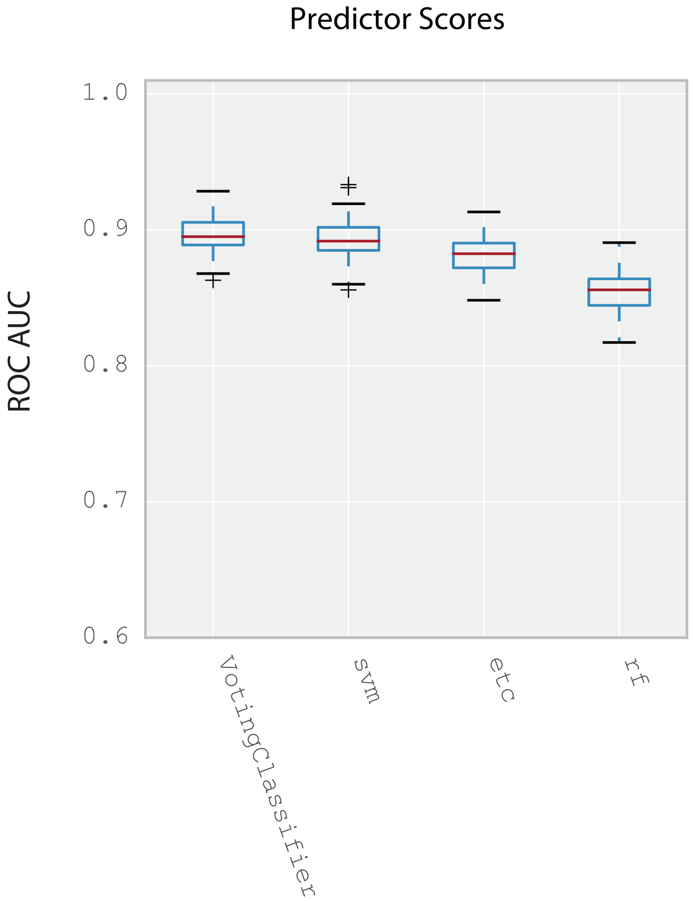
Predictor Scores
ROC AUC
1.0
0.9
0.8
0.7
0.6
VotingClassifier
svm
etc
rf

Fig S6.

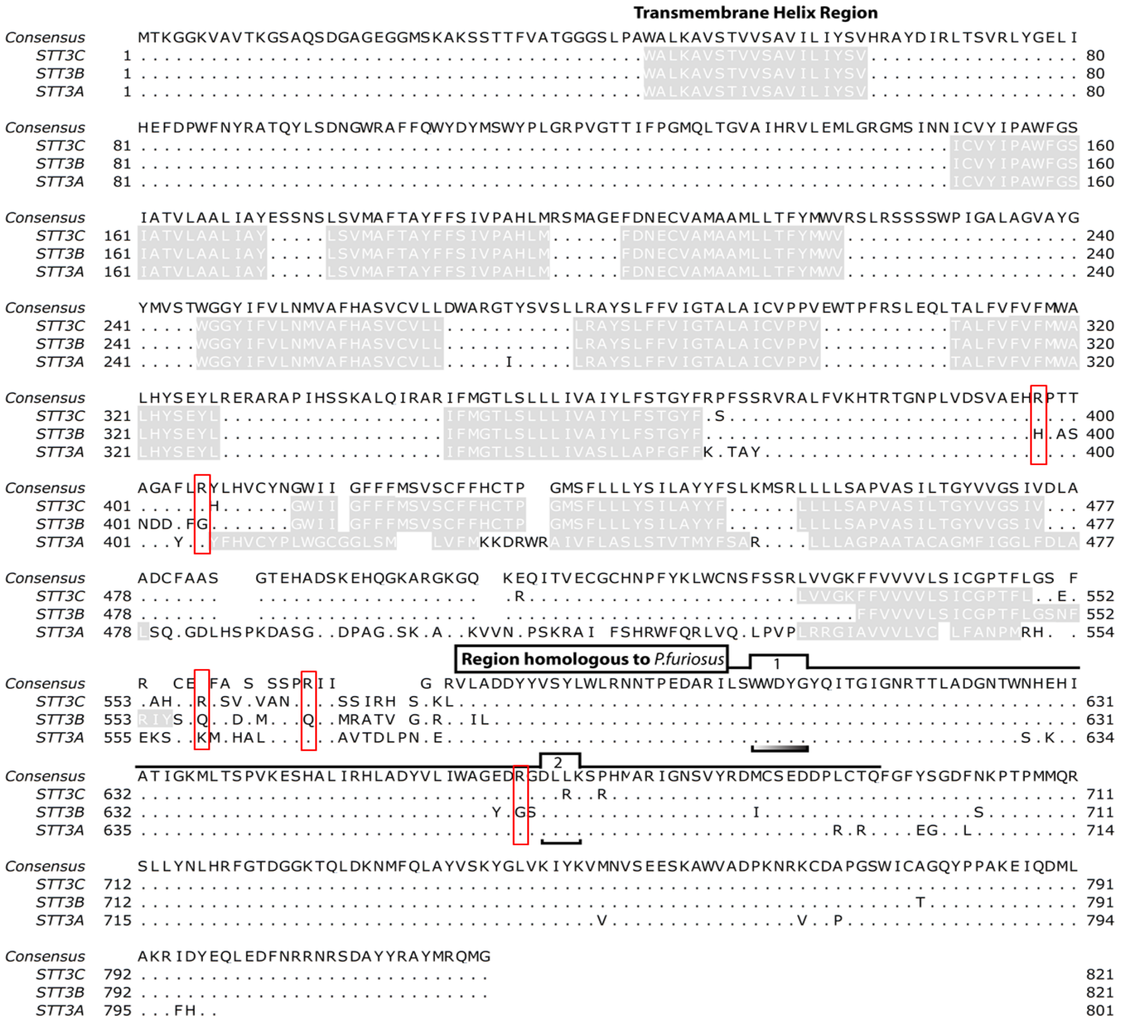

Transmembrane Helix Region
Region homologous to *P.furiosus*
1
2
Consensus
STT3C
STT3B
STT3A
